# The Use of Electronic Health Record Metadata to Identify Nurse-Patient Assignments in the Intensive Care Unit: Algorithm Development and Validation

**DOI:** 10.2196/37923

**Published:** 2022-11-09

**Authors:** Kathryn A Riman, Billie S Davis, Jennifer B Seaman, Jeremy M Kahn

**Affiliations:** 1 Department of Critical Care Medicine University of Pittsburgh School of Medicine Pittsburgh, PA United States; 2 Department of Acute & Tertiary Care University of Pittsburgh School of Nursing Pittsburgh, PA United States

**Keywords:** electronic health records, algorithms, intensive care unit, documentation, hospitals, workforce, nursing, clinical informatics, health informatics

## Abstract

**Background:**

Nursing care is a critical determinant of patient outcomes in the intensive care unit (ICU). Most studies of nursing care have focused on nursing characteristics aggregated across the ICU (eg, unit-wide nurse-to-patient ratios, education, and working environment). In contrast, relatively little work has focused on the influence of individual nurses and their characteristics on patient outcomes. Such research could provide granular information needed to create evidence-based nurse assignments, where a nurse’s unique skills are matched to each patient’s needs. To date, research in this area is hindered by an inability to link individual nurses to specific patients retrospectively and at scale.

**Objective:**

This study aimed to determine the feasibility of using nurse metadata from the electronic health record (EHR) to retrospectively determine nurse-patient assignments in the ICU.

**Methods:**

We used EHR data from 38 ICUs in 18 hospitals from 2018 to 2020. We abstracted data on the time and frequency of nurse charting of clinical assessments and medication administration; we then used those data to iteratively develop a deterministic algorithm to identify a single ICU nurse for each patient shift. We examined the accuracy and precision of the algorithm by performing manual chart review on a randomly selected subset of patient shifts.

**Results:**

The analytic data set contained 5,479,034 unique nurse-patient charting times; 748,771 patient shifts; 87,466 hospitalizations; 70,002 patients; and 8,134 individual nurses. The final algorithm identified a single nurse for 97.3% (728,533/748,771) of patient shifts. In the remaining 2.7% (20,238/748,771) of patient shifts, the algorithm either identified multiple nurses (4,755/748,771, 0.6%), no nurse (14,689/748,771, 2%), or the same nurse as the prior shift (794/748,771, 0.1%). In 200 patient shifts selected for chart review, the algorithm had a 93% accuracy (ie, correctly identifying the primary nurse or correctly identifying that there was no primary nurse) and a 94.4% precision (ie, correctly identifying the primary nurse when a primary nurse was identified). Misclassification was most frequently due to patient transitions in care location, such as ICU transfers, discharges, and admissions.

**Conclusions:**

Metadata from the EHR can accurately identify individual nurse-patient assignments in the ICU. This information enables novel studies of ICU nurse staffing at the individual nurse-patient level, which may provide further insights into how nurse staffing can be leveraged to improve patient outcomes.

## Introduction

Critical care nurses encompass the single largest workforce in the intensive care unit (ICU) and provide essential patient care 24 hours a day, 7 days a week. Adequate nurse staffing is essential for high-quality critical care; a large body of literature shows an association between patient outcomes and nurse staffing patterns, including nurse-to-patient ratios, nurse education, and nurse work environments [1-7]. This literature has been instrumental in the development of ICU staffing guidelines that strengthen ICU nursing, leading to lower mortality in US hospitals [8,9]. As beneficial as these guidelines have been, one limitation is that they focus on ICU nurses on average, rather than as individuals with varying levels of expertise, experience, and familiarity with the other members of the interprofessional care team. As a result, these approaches fail to consider the specific actions and knowledge of individual critical care nurses at the bedside and fail to account for staffing changes that occur throughout a patient’s ICU stay. These approaches are also subject to the ecological fallacy, since epidemiological relationships observed at the group level may not exist at the individual patient level [10].

More research is needed to understand how individual nurse characteristics, not just nursing characteristics in aggregate, influence patient outcomes. A critical barrier to progress in this area is the lack of a valid and reliable approach to link specific nurses to specific patients on a large scale. The electronic health record (EHR) is a potentially valuable resource for addressing this gap. Nurses use EHRs for a wide variety of tasks, including assessment documentation and medication administration. When completing these tasks, the nurse leaves behind metadata in the form of an electronic signature indicating that they were the person that performed the assessment or administered the medication. In theory, these metadata could be used to link individual nurses to specific patients during a shift, thereby generating a high-granularity measure of individual nurse-to-patient assignments. This approach would facilitate individual-level research examining the association between various nurse characteristics and patient outcomes. This research could also aid in the development of sophisticated algorithms that generate personalized nurse-to-patient assignments based on nurse skill and patient need. The objective of this study was to determine the feasibility of using the metadata from the EHR in the form of electronic signatures to determine nurse-patient assignments in the ICU.

## Methods

### Study Design and Data

**Figure 1:**
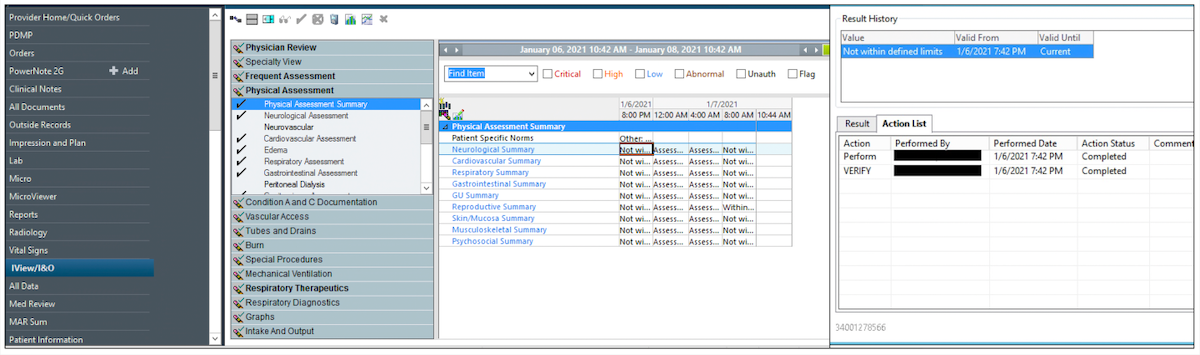
Sample screenshot of the location of nurse metadata in Cerner PowerChart. Higher-resolution version of this figure is available in Multimedia Appendix 1.

We developed and validated an algorithm for retrospectively linking individual nurses to individual patients at the level of the nursing shift. The study was conducted in a multihospital health care system in Western Pennsylvania in the United States. All hospitals shared a single enterprise-wide electronic medical record (Cerner PowerChart, Cerner Corporation) with all data warehoused in a single integrated database. All patient-level data and nurse metadata were obtained from this warehouse. To collect the data, key data elements were first identified by investigators with knowledge of the relevant clinical workflows. Relevant data were then extracted from the Cerner Millennium database (Oracle Cerner) using Cerner command language by a centralized research information technology team and transferred to the investigative team as text files (.txt) via Globus secure transfer. Data integrity was assessed for issues such as delimiter and string errors using Python (version 3.10.7; Python Software Foundation). The resulting text files were uploaded into a Microsoft SQL Server database (Microsoft Corp). Metadata of interest included date- and time-stamped electronic signatures on clinical assessments (eg, level of sedation, cardiac rhythm assessments, and neurological assessments) and medication administrations (Figure 1). Patient data included demographics, discharge disposition, as well as date and time stamps for admissions and discharges at the hospitalization and ICU-stay level. Patient data and nursing metadata were linked using direct patient identifiers.

Patients qualifying for inclusion in the analytic sample included adult patients admitted to 38 ICUs in 18 hospitals with discharge dates from January 1, 2018, to September 30, 2020. There were no specific exclusion criteria. We divided all ICU admissions between January 1, 2018, and August 31, 2020, into mutually exclusive 12-hour nursing shifts. We defined the day shift as 7 AM to 6:59:59 PM, and the night shift as 7 PM to 6:59:59 AM the following morning.

### Algorithm Development and Validation

Our algorithm has 2 main input variables from the nurse metadata: (1) the count of the number of unique times a nurse charted per patient shift and (2) the length of time between the nurse’s first and last charting times per patient shift. The count of the number of times a nurse charted per patient shift was used based on the assumption that a patient’s primary nurse would chart more frequently than other nurses. We only counted unique times instead of all charting instances because nurses could electronically sign multiple charting instances at one time. The second input variable—the length of time between the nurse’s first and last charting times per patient shift—was used based on the assumption that the primary nurse would have a longer interval between first and last charting times compared to other nurses.

**Figure 2:**
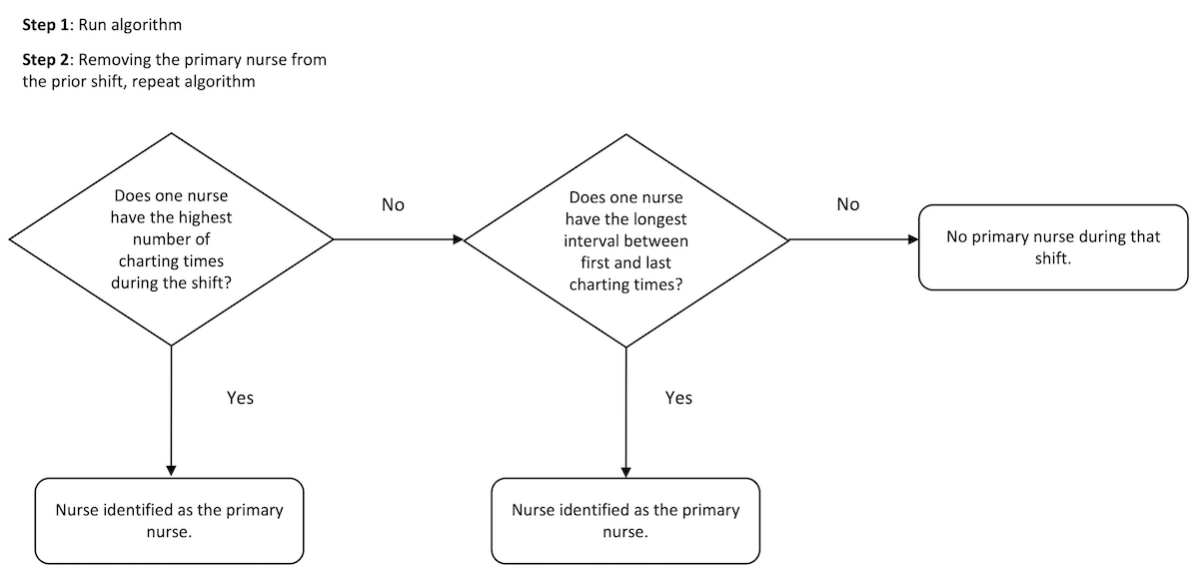
Logic model of the nurse-to-patient assignment algorithm.

Using these 2 input variables, we developed a 2-step algorithm with the following processing methods. In step 1 of the algorithm, for each patient shift, we identified the primary nurse as the nurse with the highest number of charting times during the shift, breaking ties by the longest interval between first and last charting times. If a tie remained (ie, there was more than one nurse with the same number of charting times and same charting interval), we considered there to be no primary nurse during that shift. We made this decision because we felt we could not further downselect without introducing randomness into the algorithm. In step 2, we repeated the method in step 1 but excluded the nurse from the current shift if they were the primary nurse in the prior shift (from step 1), based on the assumption that the algorithm might erroneously identify a nurse that performed an extensive amount of charting after their shift was complete. At the end of this process there were 2 output variables, as follows: (1) a binary variable indicating if each patient shift had either a primary nurse identified or not; and (2) the identity of the nurse for shifts in which a nurse was identified. Figure 2 depicts the logic model of the nurse-to-patient assignment algorithm.

To examine the underlying mechanism of the algorithm, we examined how each shift was either assigned a primary nurse or not assigned a primary nurse. Primary nurse assignment could occur in one of the following 3 ways: (1) only one nurse charted on the patient in the shift and thus had the highest number of charting times; (2) multiple nurses charted on the patient in the shift, but only one of them had the highest number of charting times; or (3) multiple nurses charted on the patient in the shift, more than one of them had the highest number of charting times, and the tie was broken based on the charting time interval. A shift could not be assigned a primary nurse also in one of the following 3 ways: (1) only one nurse charted on the patient in the shift, but it was the primary nurse in the prior shift; (2) multiple nurses charted on the patient in the shift, more than one of them had the highest number of charting times, and the tie was not broken based on the charting time interval; or (3) no nurses charted on the patient in the shift. Each shift was categorized into one of the above groups (Table 1).

We validated the performance of the nurse assignment algorithm against the reference standard of chart review. We selected a stratified random sample of 200 patient shifts, matching the proportion of patient shifts within each of the 6 categories described above. A nurse on the research team (KR) reviewed the charts, using the full range of clinical documentation to identify the actual primary nurse when such a nurse existed. We report the algorithm’s performance based on its accuracy, defined as the sum of true positives and true negatives divided by the sum of true positives, true negatives, false positives, and false negatives; and precision, defined as true positives divided by the sum of true positives and false positives. We then performed an additional review of 50 randomly selected patient shifts where no primary nurse was identified. We used this review to supplement our understanding of the reasons why no primary nurse was identified.

We described the data set and the patient sample using standard summary statistics. Precision and accuracy are reported as proportions with exact 95% CIs calculated using the binomial distribution. Data management and statistical analyses were performed using Microsoft SQL Server and Stata (version 17.0; StataCorp).

**Table 1:** Algorithm results (N=748,771).

Characteristics		Patient shifts, n (%)
**Primary nurse identified (n=728,533, 97.3%)**		
	One nurse charted	591,578 (79)
	Multiple nurses charted but one nurse charted the most times	130,591 (17.4)
	Multiple nurses charted the most times, tie broken by charting time interval	6364 (0.8)
**Primary nurse not identified (n=20,238, 2.7%)**		
	No nurse charted	14,689 (2)
	Multiple nurses charted the most times, tie not broken by charting time interval	4755 (0.6)
	One nurse charted and it was the primary nurse in the prior shift	794 (0.1)

### Ethics Approval

The University of Pittsburgh institutional review board approved the research protocol (19040420).

## Results

The final analytic data file contained 5,479,034 nurse-patient charting times; 748,771 patient-shifts; 87,466 hospitalizations; and 70,002 patients (Table 2). There were 8,134 individual nurses in the data, with 4,797 (59.0%) of them identified as the primary nurse for at least one shift. Patients had a mean age of 63.8 (SD 17.1) years; 32,199 (46.%) were female; and 58,476 (83.5%) were White. Most patients were discharged to a long-term acute care hospital or skilled nursing facility (n=36,435, 52%) or home (n=22,380, 32%; Table 3).

The algorithm performance compared to the reference standard of chart review is reported in Table 4. The algorithm was highly accurate, correctly identifying the primary nurse or correctly identifying that there was no primary nurse 93% of the time. The algorithm was also quite precise, with 94.4% of cases having the correct primary nurse when a primary nurse was identified. In the few cases where the algorithm identified one primary nurse, but chart review identified a different primary nurse, it was typically due to either an operating room or floor nurse being identified, irregular shift lengths (eg, part time nurses), or emergent scenarios (eg, cardiac arrests) in which nurses shared tasks.

In the 5 cases from the main chart review where the algorithm did not identify a primary nurse and in the 50 supplemental chart review cases, we found that the underlying cause was due to a variety of circumstances. In about half of the cases, we could identify a primary nurse in chart review. However, the information was usually in elements of the EHR not visible to the algorithm, such as transfer or discharge forms, pain assessments, or arrangements after patient death. In other cases, chart review revealed that there were 2 primary nurses, as one of the nurses was being oriented to the unit. Finally, there were some cases where no nurse was identified even via chart review because there was no digital documentation to verify the identity of the primary nurse. This often occurred when the patient was admitted to the ICU late in the shift or discharged from the ICU early in the shift, such that the time spent in the ICU was very short.

**Table 2:** Data set characteristics.

Characteristics	Values, n
Number of hospitals	18
Number of intensive care units	38
Nurse-patient charting times	5,479,034
Patient shifts	748,771
Hospitalizations	87,466
Patients	70,002
Nurses	8134
Nurses ever identified as the primary nurse	4797

**Table 3:** Patient characteristics (N=70,002).

Characteristics		Values
Hospitalizations per patient, mean (SD); (min, max)		1.2 (0.8); (1, 33)
Shifts per patient, mean (SD); (min, max)		10.7 (16.4); (1, 561)
Age (years), mean (SD); (min, max)		63.8 (17.1); (18, 119)
Sex (female), n (%)		32,199 (46.0)
**Race or ethnicity, n (%)**		
	White	58,476 (83.5)
	Black	6816 (9.7)
	Other	680 (1)
	Missing	4030 (5.8)
**Discharge disposition, n (%)**		
	Home	22,380 (32)
	Transfer to short-term hospital	2087 (3)
	Other transfer (LTAC^a^, SNF^b^)	36,435 (52)
	Died	8024 (11.5)
	Hospice	895 (1.3)
	Other or missing	181 (0.3)

^a^LTAC: long-term acute care hospital.

^b^SNF: skilled nursing facility.

**Table 4:** Algorithm performance.

Characteristics	Same or newly identified primary nurse in chart review, n	Different or no primary nurse in chart review, n
Primary nurse from algorithm	184 (true positive)	11 (false positive)
No primary nurse from algorithm	3 (false negative)	2 (true negative)

Accuracy and precision were calculated as follows:




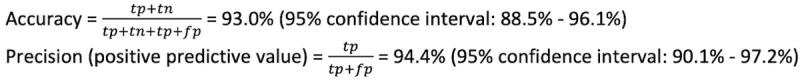




Looking back at the full data set, in the 97.3% (728,533/748,771) of patient shifts with a primary nurse identified, the median time in the ICU during the patient shift was 12 hours, compared to a median time in the ICU of 1.3 hours among the 2.7% (20,238/748,771) of patient shifts with a primary nurse not identified. In specific applications, researchers could exclude these shifts and expect an even stronger algorithm performance.

## Discussion

We developed and validated an algorithm that identifies nurse-patient assignments using metadata from the EHR. Building on a body of literature linking hospital-level measures of nurse staffing to patient outcomes [11], this study presents a novel method for characterizing individual nurse-patient assignments. This method opens several new avenues of research into the influence of nurse staffing patterns on patient outcomes. With a direct linkage of nurse to patient, it will be possible to investigate the mechanisms, nursing characteristics, and team dynamics underlying the relationship between nurse staffing and patient outcomes. Our methodology can also be applied to other roles in the care team to investigate if similar associations are present.

More broadly, this study demonstrates the potential value of EHR metadata as a tool for understanding and improving health care delivery in the ICU. Existing publicly available data sets, such as Medical Information Mart for Intensive Care, use patient data from the EHR but do not contain information at the individual provider level or link those providers to patients [12]. Registered nurses provide bedside surveillance 24 hours a day, 7 days a week and are often the first members of the care team to recognize patient deterioration. By linking individual nurses to patients, our methods progress beyond unit-wide measures of staffing and nurse characteristics and allow the generation of more granular measures to study the relationship between nurse staffing and patient outcomes.

Our work builds off prior efforts that use EHR metadata to assess health care team structure and function [13-19]. Unlike those studies, our study focused on a specific provider type and used patient care–focused metadata rather than data less tightly linked to actual care, such as the data left when an electronic chart is accessed. Informed by prior work, our method could be applied to other roles within the health care team (eg, respiratory therapists and physical therapists) to examine and optimize team dynamics and collaboration [13,15,16]. Similar to work conducted by Hribar and colleagues [14] in outpatient clinics, we may be able to examine the timing and density of tasks in the EHR to optimize scheduling of various interventions (eg, spontaneous breathing trials).

The main strengths of this study include the use of a large, multicenter data set with varying ICU types, and the innovation inherent in developing a novel yet generalizable algorithm that links nurses to patients using the EHR. Along with these strengths, this study also had several limitations that may be sources of bias or imprecision. First, the metadata we obtained were limited to EHR documentation of clinical assessments and medication administration. We focused on these domains because we considered them to be most tightly linked to clinical care, and therefore, most indicative of the actual bedside nurse. However, nurses chart other information in the EHR, and it is conceivable that using additional sources of metadata could lead to a misidentification of the bedside nurse, thereby worsening algorithm performance. Since the vast majority of shifts included relevant metadata, we suspect that any bias was minimal and overall would serve to increase the accuracy and precision of the algorithm. We also used only EHR metadata and not data from other sources, such as bed-tracking data that might directly identify the bedside nurse. Although these data may more readily allow for an accurate and precise identification of the bedside nurse, we made this decision to make our algorithm maximally generalizable, since many hospitals do not use bed-tracking software, while an increasing number of hospitals use EHRs [20]. We also chose to retain all patient shifts, not limiting to those with 12 hours in the ICU. With less time in the ICU, there is less of a chance for the primary nurse to leave behind their digital signature and a higher likelihood of misidentification (eg, assigning the ward nurse). Excluding such shifts would likely improve our algorithm performance, but we felt keeping them makes our algorithm more generalizable. Finally, the algorithm was developed using EHR data from several ICUs belonging to a large hospital system in Western Pennsylvania, which may lack generalizability to other settings and hospital systems. However, these hospitals are diverse in terms of size and academic status, making them largely representative of the US health care system.

In future work, it may be possible to apply this algorithm to other roles within the care team, such as respiratory therapists and physical therapists. Ultimately, identifying links between individual providers and individual patients will open new lines of inquiry into how provider characteristics and team characteristics are associated with individual patient outcomes. Beyond creating evidence-based nurse-to-patient assignments where the nurse’s skills are matched to the patient’s needs, we can also intentionally construct the care team to maximize care continuity and team connectedness [21,22].

In conclusion, this algorithm can accurately identify nurse-patient assignments based on nurse documentation in the EHR. This algorithm can be used by researchers to generate data on nurse-patient assignments and answer questions related to nurse health services research at the patient level and nurse assignment level.

## Appendix

Multimedia Appendix 1 Higher resolution version of Figure 1. Sample screenshot of the location of nurse metadata in Cerner PowerChart.

## Abbreviations

EHR: electronic health record
ICU: intensive care unit
