# Supplementary figures and images for: The Use of Electronic Health Record Metadata to Identify Nurse-Patient Assignments in the Intensive Care Unit: Algorithm Development and Validation
Source: JMIR Med Inform. 2022 Nov 9;10(11):e37923. doi: 10.2196/37923 (PMC9685512; doi:10.2196/37923)

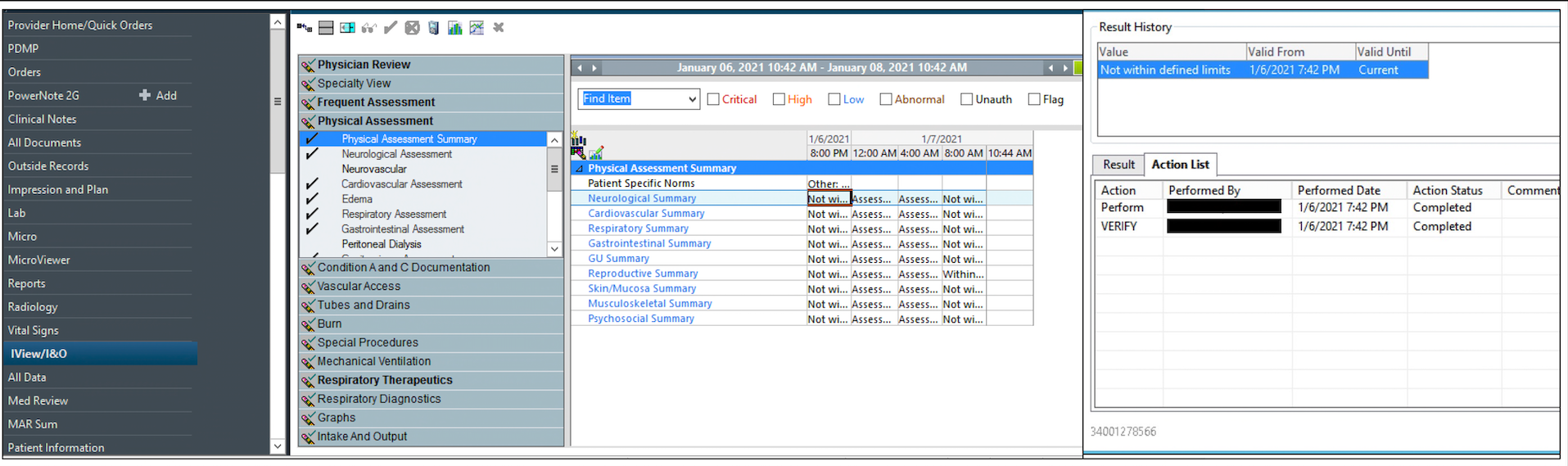

Supplement: Multimedia Appendix 1 [file medinform_v10i11e37923_app1.png]
